# Supplementary material for: Performance characteristics and potential public health impact of improved pre-erythrocytic malaria vaccines targeting childhood burden
Source: PLOS Glob Public Health. 2025 Aug 4;5(8):e0004549. doi: 10.1371/journal.pgph.0004549 (PMC12321141; doi:10.1371/journal.pgph.0004549)
Supplement: S1 Text — (DOCX) [file pgph.0004549.s001.docx]

S1 Text

**Performance characteristics and potential public health impact of improved pre-erythrocytic malaria vaccines targeting childhood burden**

*Josephine Malinga,^1,2^ Lydia Braunack-Mayer,^1,3^ Thiery Masserey,^4,5^ Aurélien Cavelan,^5^ Daniel Chandramohan,^6^ Alassane Dicko,^7^ Jean-Bosco Ouédraogo,^8^ Sherrie L Kelly,^5^ Epke A Le Rutte,^1^ Narimane Nekkab,^4,5^ Melissa A Penny^1,2 *^*

^1^ The Kids Research Institute Australia, Nedlands, Western Australia, Australia

^2^ Centre for Child Health Research, University of Western Australia, Crawley, Western Australia, Australia

^3^ Institute of Social and Preventive Medicine, University of Bern, Bern, Switzerland

^4^ Department of Epidemiology and Public Health, Swiss Tropical and Public Health Institute, Allschwil, Switzerland

^5^ University of Basel, Basel, Switzerland

^6^ London School of Hygiene & Tropical Medicine, London, United Kingdom

^7^ Malaria Research and Training Centre, University of Sciences, Techniques, and Technologies of Bamako, Bamako, Mali.

^8^ Institut des Sciences et Techniques-Institut de Recherche en Sciences de la Santé, Bobo-Dioulasso, Burkina Faso

*Corresponding author

Prof Melissa A Penny

**melissa.penny@uwa.edu.au**

Table of Contents

[1. **Materials and Methods 2**](#_Toc187228807)

[a. Mathematical transmission model 2](#_Toc187228808)

[b. Vaccine deployment in settings with constant transmission 5](#_Toc187228809)

[**2. Validation of vaccine and chemoprevention properties using clinical trial data 5**](#_Toc187228810)

[a. Trial characteristics 5](#_Toc187228811)

[b. Vaccine decay profile and seasonality profiles 7](#_Toc187228812)

[c. Modelled scenarios and trial endpoints 7](#_Toc187228813)

[d. Bayesian optimization approach 8](#_Toc187228814)

[e. Bayesian optimization results for all arms in the two countries 8](#_Toc187228815)

[f. Additional in silico modelling results 9](#_Toc187228816)

[**3. Additional modelling results 10**](#_Toc187228817)

[a. Public health impact of improved PEVs on the malaria burden 10](#_Toc187228818)

[b. Drivers of impact following a five-year vaccination program 14](#_Toc187228819)

[c. Multi-year vaccine impact in the two years following primary vaccination 17](#_Toc187228820)

1. Materials and Methods
2. Mathematical transmission model

OpenMalaria is an individual based stochastic simulation model of malaria in humans linked to a deterministic model of malaria in mosquitoes [1–3]. The details on model structure and fitting process have been described in full [4]. The open-source code is available on (https://github.com/SwissTPH/openmalaria/wiki), including detailed documentation. At its core, OpenMalaria captures the clinical epidemiology and natural history of malaria and includes sub-models of infection in humans, blood stage parasite dynamics, infectiousness to mosquitoes and incidence of mortality and morbidity, and immunity. The model also accounts for genetic diversity, enabling the study of drug resistance, vaccine insensitivity, and vector susceptibility. All models have been fitted to field data from malaria endemic areas, as described in previous studies as shown in Table A. We provide an overview of OpenMalaria as used in our study.

In summary, malaria infection in humans is driven by the entomological inoculation rate (EIR) which affects the force of infection. Infection events and outcomes, parasite densities, mosquito and human elements are updated at each 5-day timesteps. Blood-stage parasite density depends on the time since infection and is affected by naturally acquired immunity. Acquired immunity, which reduces parasite density of subsequent infections, develops progressively following repeated exposure to infections and total parasitemia. Acute clinical illness depends on human host parasite densities and their pyrogenic threshold which evolves over time depending on previous exposure. Morbidity episodes can be uncomplicated or evolve to severe disease where a proportion of these severe cases leads to death. Transmission to mosquitoes depends on the parasite density present in the humans and a time-lag to allow the development of gametocytes. The overall infectiousness to mosquitoes is age-specific and has been validated to field data. OpenMalaria is also composed of a comprehensive simulation of the mosquito lifecycle and behavior towards human and animal hosts, including biting rates, resting and survival probabilities, embedded in a dynamic entomological model of the mosquito feeding cycle. All through the human and mosquito stages, the effect of various interventions on preventing infection, clinical episodes or onward transmission such as vector control, case management and vaccines can be modelled. The full characteristics of the model, modelled setting characteristics and entomological factors, outcome definitions are summarized in Table A and Table B.

**Table A: Summary of OpenMalaria components and properties adapted from Golumbeanu [5]**

| Name | Description | References |
| --- | --- | --- |
| ***Key epidemiological processes*** | | |
| Malaria infection in humans | - Driven by the entomological inoculation rate (EIR) which is a model input and affects the force of infection in the simulated settings - Human exposure to mosquitoes depends on age and is corelated with the body surface area | [6] |
| Progression of infections in humans: parasite densities and immunity | - Several sub-models of asexual parasite densities are included within OpenMalaria. In each model, blood-stage parasite density depends on the time since infection and is affected by naturally acquired immunity. - Acquired immunity (both pre-erythrocytic and blood-stage) develops progressively following repeated exposure to infections and total parasitemia. Acquired immunity reduces parasite density of subsequent infections. - The duration of infection follows a log-normal distribution and is estimated from a malaria therapy dataset. - Super-infection (multiple distinct infections) is possible with cumulative parasite densities | [1,6–8] |
| Transmission from infected humans to mosquitoes | - Transmission to mosquitoes depends on the parasite density present in the humans (including a time-lag for gametocyte development) - The age-specific contribution to overall infectiousness to mosquitoes was validated in against field data in malaria endemic countries. | [1,9,10] |
| Clinical illness, morbidity, and mortality | - Acute clinical illness depends on human host parasite densities and their pyrogenic threshold which evolves over time depending on the individual exposure history - Acute morbidity episodes can be uncomplicated or evolve to severe episodes - A proportion of the severe episodes leads to deaths | [1,11–13] |
| ***Modelled characteristics of the transmission settings*** | | |
| Population age structure | - Informed by health and demographic surveillance data from Ifakara, Tanzania | [11,14] |
| Transmission seasonality | - Seasonally forced, the same transmission pattern is reproduced each year in absence of interventions. - Seasonal patterns are inputs to the model and users can define any patterns as needed e.g., perennial, one or two peak seasonal patterns, etc. | [6,15] |
| Entomological setting | - Comprehensive simulation of the mosquito lifecycle and behavior towards human and animal hosts (biting, resting) embedded in a dynamic entomological model of the mosquito feeding cycle - Multiple vector species can be simulated simultaneously | [3] |
| Case Management | - Modelled through a comprehensive decision tree-based model which determines the corresponding treatment implications depending on the occurring clinical events such as fevers and seeking of care - Its representation includes specification of access to official or nonofficial care, access to hospital for severe cases, diagnostic tests (use, specificity, sensitivity, and threshold of detection), treatments for first, second line and non-official care, effects of treatment, case fatality rate, case sequelae and cure rates | [16] |
| ***Modelled interventions*** | | |
| Vector control | - Vector interventions can deter mosquitoes from humans, reduce the probability that a mosquito bites a protected human host or reduce the probability that a mosquito survives host feeding. - These include bed nets, indoor residual spraying, repellents e.t.c | [17–19] |
| Drugs and Vaccines | - Drugs and vaccines act at different stages of the parasite life cycle (transmission blocking, anti-infective, blood-stage clearance) and their action is defined by their initial efficacy against infection, half-life, and decay | [20,21] |
| Deployment characteristics | - Interventions can be deployed for several rounds to a targeted group of individuals and specified coverages (proportion of the population reached with the intervention) - Interventions can be deployed by enrolling individuals into cohorts and tracking cohort outcomes, facilitating clinical trial simulation |  |
| ***Simulation regimens and model variants*** | | |
| Time Steps | - Simulation outputs are tracked every 5 days (one time step) |  |
| Model variants | - Varying assumption on treatment, immunity decay, heterogeneity of transmission etc results in 14 model variants. In the current study, we only use the base model. | [22] |
| ***Software availability and documentation*** | | |
| Source code and wiki page available on GitHub: https://github.com/SwissTPH/openmalaria/ | | |

**Table B: Simulated scenarios and outcome measures**

| **Parameter** | **Description** |
| --- | --- |
| ***Primary Outcomes*** | |
| Infection prevalence reduction | The relative reduction in infection prevalence compared with a no intervention counterfactual, where prevalence was defined as the proportion of all malaria infections (detectable by a highly sensitive diagnostic such as PCR) across the six-, 12-, or 24-month period after the final vaccine booster dose in the fifth year, or in six-monthly intervals until 18 months after primary series doses |
| Clinical incidence reduction | The relative reduction in the incidence of clinical cases compared with a no intervention counterfactual, where incidence was defined as the number of new, uncomplicated malaria cases across the six-, 12-, or 24-month period after the final vaccine booster dose in the fifth year, or in six-monthly intervals until 18 months after primary series doses  Uncomplicated malaria was defined as an episode of symptomatic malaria, detectable by rapid diagnostic test with 94.2% specificity and a detection limit of 50 parasites per microlitre, where symptoms did not qualify as severe malaria. |
| Severe malaria cases reduction | The relative reduction in the incidence of severe malaria cases compared with a no intervention counterfactual, where severe cases of malaria were evaluated across the six-, 12-, or 24-month period after the final vaccine booster dose in the fifth year, or in six-monthly intervals until 18 months after primary series doses.  An episode of severe malaria was an episode of hospitalized symptomatic malaria, with symptoms qualifying as severe malaria or with co-morbidities |
| ***Target population*** | Vaccine impact was evaluated for two target populations   - Children under five years old - Children under three years old for multi-seasonal impact in the first and second years after vaccination |
| ***Setting characteristics*** | |
| *Pf*PR_2-10_ | Baseline parasite prevalence before intervention of between 10% and 50% |
| Seasonality | Highly seasonal (4-month profile), Seasonal (6-month profile) and constant transmission |
| Mosquito species | *Anopheles gambiae* |
| ***Health system characteristics*** | |
| Access to care | Probability of accessing effective curative malaria treatment within 14-days of symptomatic malaria. Treatment is assumed to clear all blood stage infections within 5 days   - Low probability of 10% - High probability of 70% |
| Diagnostic | Rapid diagnostic tests (RDTs) |

1. Vaccine deployment in settings with constant transmission

**Fig A: Schematic illustration of the simulated vaccine deployment schedules for a five-year vaccination program shown for a setting with constant transmission.**

Illustration shows the five-year vaccination program with a primary series dosage, the timing of the annual boosters and the age groups at each dose for both vaccine delivery schedules, hybrid vaccination (grey boxes) and mass vaccination (brown boxes). For the mass vaccination schedule, the primary series and annual booster doses are deployed before the peak transmission season. For the hybrid vaccination schedule, doses for the primary series are deployed continuously as part of an age-based immunization schedule while the annual boosters are also deployed before the peak transmission season. The green shading shows the zero to 18 months period where the multi-seasonal or multi-year vaccine impact is evaluated by comparing a cohort of children who received the primary series doses and the first booster (dose four) and those who only received the primary series doses. The blue shading shows the 12-month period following the final annual booster dose (dose seven) in the fifth year where the public health vaccine impact is evaluated for children who received the primary series followed by annual boosters.

1. Validation of vaccine and chemoprevention properties using clinical trial data
2. Trial characteristics

We used results from a recently conducted Phase 3 clinical trial for seasonal vaccination with RTS,S in Mali and Burkina Faso to validate the vaccine properties of RTS,S. Specifically, we validated the vaccine’s efficacy against infection reached after boosting in the two years following primary vaccination. This comparison informed model assumptions around the efficacy profile of a first booster/fourth dose given 12 months after the three-dose primary series, rather than 18 months after, as implemented in the original Phase 3 clinical trial conducted in seven sites in African countries and a second booster/fifth dose given 24 months after. We validate the results for the clinical trial arms, including: seasonal vaccination using RTS,S alone, seasonal vaccination using RTS,S given in combination with seasonal chemoprevention using SPAQ, and seasonal chemoprevention using SPAQ alone. Similarly, using a similar approach described and the results from the same Phase 3 clinical trial in Mali and Burkina Faso we calibrate the preventive half-life duration of seasonal chemoprevention using SPAQ.

We compared our model results across two seasonal transmission profiles with seasonality patterns taken from Mali and Burkina Faso (Fig B). We selected the baseline EIR translating to a *Pf*PR_2-10_ between 5-65% and *Pf*PR_6-12_ between 10-70% to reproduce the malaria epidemiology in the two study sites. We also modelled other interventions including vector control. Each trial participant was given a long-lasting insecticide at the beginning of the trial. Any other interventions not reported as part of the trial were assumed to remain constant and are reflected in the baseline EIR. We assume a 45% probability of accessing curative malaria treatment within 14 days of the onset of symptoms. We reproduce the clinical trial by recruiting a cohort of children aged five to 17 months and following them up for two years, as done in the clinical trial.

In a second analysis evaluating the duration of protection in the aforementioned seasonal chemoprevention and vaccination trial in Mali and Burkina Faso, the 6-monthly vaccine protective efficacy amongst children receiving SMC was at 75.9% [95% CI 67.0–82.4%] in the first year after the three-dose primary vaccination series, 63.6% [95% CI 57.2–69.1%] in the second year after the first booster/fourth dose, and 60.1% [95% CI 52.9–66.2%] in the third year after the second booster/fourth dose. This analysis showed wider confidence intervals after the initial six to seven months (~200 days) following vaccination in each year reflecting a biphasic decay profile with a long tail [23,24]. This translates to between 10% to 30% drop in protective efficacy against clinical disease in years two and three compared with year one, in both countries [23,24].

**Table C: Trial characteristics matched to modelled input to check intervention parameterization**

| **Parameter** | **OM parameter** | **Trial** | **Reference** |
| --- | --- | --- | --- |
| Mosquito species | *Anopheles gambiae*: 80% indoor biting and 20% outdoor biting |  |  |
| Entomological inoculation rate (EIR)^*^ | Mali: EIR to match *Pf*PR_6-12_ = ~20%  Burkina Faso: EIR to match *Pf*PR_6-12_ = ~60% |  |  |
| Seasonality | Mali: Highly seasonal transmission  Burkina Faso: Seasonal transmission |  |  |
| Malaria transmission intensity^*^ (as *PfPR_6-12_ measured towards end of transmission season*) | Mali: *Pf*PR_6-12_ = ~20%  Burkina Faso: *Pf*PR_6-12_ = ~60% | Mali: *Pf*PR_6-12_ = ~20%  Burkina Faso: *Pf*PR_6-12_ =~60% | [23] |
| Access to treatment | Effective coverage over 14-days of symptom onset of 45% represented in OpenMalaria as 20% over 5-day time-steps | High access to case management following symptom onset (trial level) |  |
| Clinical malaria treatment pathway | First-line treatment with artemether-lumefantrine; Second-line treatment with quinine |  |  |
| Insecticide treated net coverage | Assume all children in the trial are covered by a long-lasting insecticide net | All children received a long-lasting insecticide net at the beginning of the trial |  |
| Vaccine timing | Mali: April to July 2017 (primary series), booster doses June 2018 and 2019  Burkina Faso: May to July 2017 (primary series), booster doses June 2018 and 2019 | Mali: April to July 2017 (primary series), booster doses June 2018 and 2019  Burkina Faso: May to July 2017 (primary series), booster doses June 2018 and 2019 | [23] |
| SMC timing | Mali: July to October  Burkina Faso: July to October |  | [23] |

*^*^Pf*PR_6-12_: *Plasmodium falciparum* parasite rate in 6- to 12-year-olds

1. Vaccine decay profile and seasonality profiles

**Fig B: Schematic illustration of vaccine decay profile over two years and the yearly seasonal profiles used as model input for Burkina Faso and Mali.**

A) Illustration shows a two- year vaccine efficacy against infection decay profile following the three dose primary series and the first annual booster given in the first year. The initial efficacy against infection reached after dose three is 91.1%, a half-life duration of 7.32 months with a biphasic decay with shape parameter k = 0.69. The booster dose is assumed to follow the same decay profile. The light grey-blue solid line represents the booster, a dashed darker grey-blue line represents the scenario without booster. B) Yearly seasonal profiles used as model inputs for Burkina Faso and Mali.

1. Modelled scenarios and trial endpoints

Modelled estimates from the original Phase 3 clinical trial conducted in seven sites in African countries [25,26], show RTS,S to have a high initial vaccine efficacy against infection of 91.1% [95% CI 74.5–99.7%] following the three-dose primary vaccination, with a biphasic immunity decay profile where the shape parameter was k = 0.69, and a half-life duration (time until initial efficacy against infection decays by 50%) was 7.32 months [95% CI 6–10 months] for children aged 5-17 months at recruitment. Vaccine efficacy against infection could be restored to 49.9% [95% CI 32.0–68.6%] following a single booster dose given 18 months after the three-dose primary vaccination [25].

In this study, the previously reported estimates for initial efficacy against infection after primary vaccination, decay function and half-life duration of protection are used as model inputs. To estimate the vaccine initial efficacy against infection reached after the first and second boosters, we assume that the boosting efficacy against infection falls by between 10% to 20% after the first booster/fourth dose, and 20% to 30% after the second booster/fifth dose. Assumptions on parameter ranges, modelled for boosting efficacy against infection and the decline in protection during the second and third years in this study, were based on previous clinical and modelling evidence for RTS,S [23–25,27], as described above. Vaccine efficacy against infection decays with a biphasic exponential function [25,28], declining rapidly in the initial six to seven months after the primary vaccination series followed by a slow decay with a long tail. The same decay profile is assumed for the primary series and following the booster doses.

To match the clinical trial endpoints, we first extracted the monthly clinical incidence over the three-year trial period and we used in the model fitting. We also calculated the cumulative incidence and the hazard ratios for both countries separately and combined to reproduce the published estimates [23]. For this modelling study, we only focus on the primary outcome as reported from the clinical trial: uncomplicated clinial episodes.

1. Bayesian optimization approach

We used a bayesian optimization (BO) approach, to fit our model results to clinical trial data, providing the best fit values for both countries for all the three trial arms. BO is an approach used for hyperparameter optimization in machine learning to find the global optima with a limited number of function evaluations [29]. BO combines a probabilistic model of the objective function, in this case a Gaussian Process (GP) model, with an acquisition function to minimize a loss function. For this study, we integrated BO with PyTorch and the botorch library[30], a framework for deep learning which allowed for efficient computation [29]. We used the residual sum of squares (RSS) as the loss function to ensure improved regression model accuracy. The RSS is a measure of the discrepancy between observed and the predicted values, in this case the clinical trial data and modelled results, and is defined as:

$$RSS= \sum_{i=1}^{n} \left( y^{i}- \hat{y}^{i} \right)^{2}$$

where $y^{i}$ represents the observed values and $\hat{y}^{i}$ represents the predicted values.

We used a GP as the surrogate model to provide a probabilistic estimate of the objective function and used the Expected Improvement (EI) as the acquisition function [29,31]. EI quantifies the expected improvement over the current best observation guiding the search towards regions with a high probability of improvement, hence balancing exploration of uncertainty and exploitation of the most promising points to evaluate next. The function returns the best candidate observations given the mean and variance of the surrogate model. The implementation began with initializing a set of training points with synthetic data used to fit the initial GP model. During each iteration, the EI acquisition function was optimized to identify the most likely points for evaluation in the subsequent step. These points were then evaluated using the RSS and the outcomes added to the training set, hence, updating the GP model iteratively. For each trial arm, the process was replicated for 1000 iterations progressively refining the search space for the optimal parameters that minimize the RSS. To help quantify the uncertainty in the final solution and summarize the optimization results, the 95% confidence intervals (CIs) were computed from the optimal values with the minimum RSS.

1. Bayesian optimization results for all arms in the two countries

Table D: Bayesian optimization results for all parameters across the three trial arms

| **Arm** | **Vaccine alone arm** | **SMC alone arm** | **Combined arm** |
| --- | --- | --- | --- |
| **Prevalence^*^**   - **Burkina Faso** - **Mali** | *Pf*PR_6-12_ = ~60%  *Pf*PR_6-12_ = ~20% | *Pf*PR_6-12_ = ~60%  *Pf*PR_6-12_ = ~20% | *Pf*PR_6-12_ = ~60%  *Pf*PR_6-12_ = ~20% |
| **Access to care within two weeks of symptom onset** | 45% | 45% | 45% |
| **Dose 4 boosting efficacy against infection** | 78.96%  [95%CI 70.74 – 87.18%] |  | 77.45%  [95%CI 68.26 – 86.62%] |
| **Dose 5 boosting efficacy against infection** | 57.31%  [95%CI 44.05 – 70.56%] |  | 48.76%  [95%CI 34.58 - 65.95%] |
| **SMC (SPAQ half-life duration of protection)** |  | 24.6 days  [95%CI 21.3 - 27.9] | 25.3 days  [95%CI 22.9 – 27.7] |
| **SMC (SPAQ decay of protection, Weibull parameter (k))** |  | 4.05  [95%CI 3.33 – 4.76] | 3.82  [95%CI 2.92 – 4.71] |
| **Other interventions** | ITN | ITN | ITN |

*^*^Pf*PR_6-12_: *Plasmodium falciparum* parasite rate in 6- to 12-year-olds

1. Additional in silico modelling results

**Fig C: Monthly clinical incidence for the clinical trial data (black dots) compared with model simulations (blue lines/triangles) using the best fit assumption for the efficacy against infection of annual booster doses four and five and SPAQ preventive half-life duration.** Best fit parameters for Burkina Faso (high prevalence and seasonal transmission), Mali (moderate prevalence and highly seasonal transmission) and both countries aggregated shown for the three trial arms. The black dots shown with 95% confidence intervals represent the trial field data and the blue lines/triangles illustrate the modelled output from the simulations with the shaded region showing the confidence intervals averaged over 100 seeds. In this figure, the parameters were optimized for the arm where the vaccine and chemoprevention were combined and used to predict the model results for the arms where chemoprevention or vaccination were given alone.

**Fig D: Hazard Ratios the clinical trial data (black dots) compared with model simulations (blue lines/triangles) using the best fit assumption for the efficacy against infection of annual booster doses four and five and SPAQ preventive half-life duration.** Hazard ratios for the three trial arms in Burkina Faso (high prevalence and seasonal transmission), Mali (moderate prevalence and highly seasonal transmission) and both countries combined. The black dots shown with 95% confidence intervals represent the trial field data and the blue lines/triangles illustrate the modelled output from the simulations with the shaded region showing the confidence intervals averaged over 100 seeds. In this figure, the parameters were optimized for the arm where the vaccine and chemoprevention were combined and used to predict the model results for the arms where chemoprevention or vaccination were given alone.

1. Additional modelling results
2. Public health impact of improved PEVs on the malaria burden

**Fig E: Predicted relative burden reduction in the 12-month period following the final annual booster dose, compared with a no-intervention counterfactual.**

A) Target reduction (%) in infection prevalence (top row), clinical incidence (middle row), and severe disease (bottom row) illustrating trade-offs between initial efficacy against infection and half-life duration of protection in settings where baseline *Pf*PR_2-10_ ranged between 10% and 20%. The initial efficacy against infection ranged from 50% to 100%, half-life duration from six to 18 months, and assuming a primary series vaccination coverage of 90%. B) Median (interquartile range (IQR)) relative reduction in infection prevalence (top row), clinical incidence (middle row), and severe disease (bottom row), considering varying levels of baseline *Pf*PR_2-10_, coverage, and initial efficacy against infection for a long duration vaccine with a half-life duration between 12 and 18 months. Results are shown for a setting where the PEV was not co-administered with a blood stage clearance drug, for a four-month short seasonality profile, for the mass vaccination deployment schedule, in settings with a 30% probability of accessing curative treatment within 14 days of symptom onset.

**Fig F: Predicted relative burden reduction in the 12-month period following the final annual booster dose, compared with a no-intervention counterfactual.**

A) Target reduction (%) in infection prevalence (top row), clinical incidence (middle row), and severe disease (bottom row) illustrating trade-offs between initial efficacy against infection and half-life duration of protection in settings where baseline *Pf*PR_2-10_ ranged between 10% and 20%. The initial efficacy against infection ranged from 50% to 100%, half-life duration from six to 18 months, and assuming a primary series vaccination coverage of 90%. B) Median (interquartile range (IQR)) relative reduction in infection prevalence (top row), clinical incidence (middle row), and severe disease (bottom row), considering varying levels of baseline *Pf*PR_2-10_, coverage, and initial efficacy against infection for a long duration vaccine with a half-life duration between 12 and 18 months. Results are shown where the PEV was co-administered with a blood stage clearance drug, for a 6-month long seasonality profile and constant transmission, for the mass vaccination deployment schedule, in settings with a 30% probability of accessing curative treatment within 14 days of symptom onset.

**Fig G. Predicted relative reduction in infection prevalence, clinical incidence and severe disease for the 12-month period following the final annual booster dose, compared with a no-intervention counterfactual.**

A) Target reduction (%) in infection prevalence (top row), clinical incidence (middle row), and severe disease (bottom row) illustrating trade-offs between initial efficacy against infection and half-life duration of protection in settings where baseline *Pf*PR_2-10_ ranged between 10% and 20%. The initial efficacy against infection ranged from 50% to 100%, half-life duration from six to 18 months, and assuming a primary series vaccination coverage of 90%. B) Median (interquartile range (IQR)) relative reduction in infection prevalence (top row), clinical incidence (middle row), and severe disease (bottom row), considering varying levels of baseline *Pf*PR_2-10_, coverage, and initial efficacy against infection for a long duration vaccine with a half-life duration between 12 and 18 months. Results are shown for a PEV co-administered with a blood stage clearance drug, for a four-month short seasonality profile, for the mass vaccination deployment schedules, in settings with a moderate (30%) and high (70%) probability of accessing curative treatment within 14 days of symptom onset.

**Fig H: Predicted relative reduction in infection prevalence, clinical incidence and severe disease for the 12-month period following the final annual booster dose, compared with a no-intervention counterfactual.**

A) Target reduction (%) in infection prevalence (top row), clinical incidence (middle row), and severe disease (bottom row) illustrating trade-offs between initial efficacy against infection and half-life duration of protection in settings where baseline *Pf*PR_2-10_ ranged between 10% and 20%. The initial efficacy against infection ranged from 50% to 100%, half-life duration from six to 18 months, and assuming a primary series vaccination coverage of 50%. B) Median (interquartile range (IQR)) relative reduction in infection prevalence (top row), clinical incidence (middle row), and severe disease (bottom row), considering varying levels of baseline *Pf*PR_2-10_, coverage, and initial efficacy against infection for a short duration vaccine with a half-life duration between six and 12 months. Results are shown for a PEV co-administered with a blood stage clearance drug, for a four-month short seasonality profile, for both deployment schedules, in settings with a 30% probability of accessing curative treatment within 14 days of symptom onset.

1. Drivers of impact following a five-year vaccination program

**Fig I: Factors influencing vaccine impact on predicted burden reductions for the 12-month period following the final annual booster dose compared with a no-intervention counterfactual.**

A) The bars represent the total Sobol effect indices which quantify the variance in predictions of relative reduction. These indices can be interpreted as the proportion of variation in the outcome attributed to changes in each variable. Results are shown across various baseline *Pf*PR_2-10_ values and span different parameter ranges for initial efficacy against infection (70% to 100%), half-life duration (six to18 months), and vaccination coverage (60% to 90%). B) The influence of the impact-driving factors on predicted reductions in infection prevalence and clinical incidence for settings where *Pf*PR_2-10_ lies between 20% and 30%. The different lines and shaded areas depict the median and interquartile range (IQR) of proportional contribution, as estimated through global sensitivity analysis over the variable parameter ranges for initial efficacy against infection (60% to 100%), half-life duration (six to 18 months) and vaccination coverage of (60% to 95%). Results are shown for a setting where the PEV was not co-administered with a blood stage clearance drug, for a four-month short seasonality profile, for the mass vaccination deployment schedules, in settings with a 30% probability of accessing curative treatment within 14 days of symptom onset.

**Fig J: Factors influencing vaccine impact on predicted burden reductions for the 12-month period following the final annual booster dose compared with a no-intervention counterfactual.**

A) The bars represent the total Sobol effect indices which quantify the variance in predictions of relative reduction. These indices can be interpreted as the proportion of variation in the outcome attributed to changes in each variable. Results are shown across various baseline *Pf*PR_2-10_ values and span different parameter ranges for initial efficacy against infection (70% to 100%), half-life duration (six to18 months), and vaccination coverage (60% to 90%). B) The influence of the impact-driving factors on predicted reductions in infection prevalence and clinical incidence for settings where *Pf*PR_2-10_ lies between 20% and 30%. The different lines and shaded areas depict the median and interquartile range (IQR) of proportional contribution, as estimated through global sensitivity analysis over the variable parameter ranges for initial efficacy against infection (60% to 100%), half-life duration (six to 18 months) and vaccination coverage of (60% to 95%). Results are shown where the PEV was co-administered with a blood stage clearance drug, for a 6-month long seasonality profile and constant transmission, for the mass vaccination deployment schedule, in settings with a 30% probability of accessing curative treatment within 14 days of symptom onset.

**Fig K: Factors influencing vaccine impact on predicted burden reductions for the 12-month period following the final annual booster dose compared with a no-intervention counterfactual.**

A) The bars represent the total Sobol effect indices which quantify the variance in predictions of relative reduction. These indices can be interpreted as the proportion of variation in the outcome attributed to changes in each variable. Results are shown across various baseline *Pf*PR_2-10_ values and span different parameter ranges for initial efficacy against infection (70% to 100%), half-life duration (six to18 months), and vaccination coverage (60% to 90%). B) The influence of the impact-driving factors on predicted reductions in infection prevalence and clinical incidence for settings where *Pf*PR_2-10_ lies between 20% and 30%. The different lines and shaded areas depict the median and interquartile range (IQR) of proportional contribution, as estimated through global sensitivity analysis over the variable parameter ranges for initial efficacy against infection (70% to 100%), half-life duration (six to 18 months) and vaccination coverage of (60% to 90%). Results are shown for a PEV co-administered with a blood stage clearance drug, for a four-month short seasonality profile, for the mass vaccination deployment schedules, in settings with a moderate (30%) and high (70%) probability of accessing curative treatment within 14 days of symptom onset.

1. Multi-year vaccine impact in the two years following primary vaccination

**Fig L: Predicted relative reduction and impact-drivers on clinical incidence in the zero to 24 months following primary vaccination for children who did not receive a booster compared to a no-intervention counterfactual.**

A) Median (interquartile range (IQR)) relative reduction in clinical incidence for different levels of *Pf*PR_2-10_, two levels of initial efficacy against infection (50%, 95%), for a long duration vaccine with a half-life between 12 and 18 months and primary series vaccination coverage of 100%. B) Trade-offs between initial efficacy against infection and half-life duration of protection for clinical incidence reduction in settings where baseline *Pf*PR_2-10_ ranged between 10% and 20%, initial efficacy against infection ranged from 50% to 100%, and half-life duration from six to 18 months, assuming a primary series vaccination coverage of 100%. C) The bars represent the total Sobol effect indices which quantify the variance in predictions of relative reduction in clinical incidence. These indices can be interpreted as the proportion of variation in the outcome attributed to changes in each variable. Results are shown over different parameter ranges for initial efficacy against infection (75% to 100%) and half-life duration (six to18 months), where vaccination coverage was fixed at 100%. Results are shown for a PEV co-administered with a blood stage clearance drug, for the zero- to six-, six- to 12-, 12- to 18- and 18- to 24-month periods following primary vaccination, for a six-month short seasonality profile, the hybrid vaccination schedule, in settings with a 30% likelihood of accessing curative treatment within 14 days of symptom onset.

**Fig M: Predicted relative reduction and impact-drivers on clinical incidence in the zero to 24 months following primary vaccination for children who received a booster compared to a no-intervention counterfactual.**

A) Median (interquartile range (IQR)) relative reduction in clinical incidence for different levels of *Pf*PR_2-10_, two levels of initial efficacy against infection (50%, 95%), for a long duration vaccine with a half-life between 12 and 18 months and primary series vaccination coverage of 100%. B) Trade-offs between initial efficacy against infection and half-life duration of protection for clinical incidence reduction in settings where baseline *Pf*PR_2-10_ ranged between 10% and 20%, initial efficacy against infection ranged from 50% to 100%, and half-life duration from six to 18 months, assuming a primary series vaccination coverage of 100%. C) The bars represent the total Sobol effect indices which quantify the variance in predictions of relative reduction in clinical incidence. These indices can be interpreted as the proportion of variation in the outcome attributed to changes in each variable. Results are shown over different parameter ranges for initial efficacy against infection (75% to 100%) and half-life duration (six to18 months), where vaccination coverage was fixed at 100%. Results are shown for a PEV co-administered with a blood stage clearance drug, for the zero- to six-, six- to 12- , 12- to 18- and 18- to 24-month periods following primary vaccination, for a six-month short seasonality profile, for the mass vaccination schedule, in settings with a 30% likelihood of accessing curative treatment within 14 days of symptom onset.

**Fig N: Predicted relative reduction and impact-drivers on clinical incidence in the zero to 24 months following primary vaccination for children who did not receive a booster compared to a no-intervention counterfactual.**

A) Median (interquartile range (IQR)) relative reduction in clinical incidence for different levels of *Pf*PR_2-10_, two levels of initial efficacy against infection (50%, 95%), for a long duration vaccine with a half-life between 12 and 18 months and primary series vaccination coverage of 100%. B) Trade-offs between initial efficacy against infection and half-life duration of protection for clinical incidence reduction in settings where baseline *Pf*PR_2-10_ ranged between 10% and 20%, initial efficacy against infection ranged from 50% to 100%, and half-life duration from six to 18 months, assuming a primary series vaccination coverage of 100%. C) The bars represent the total Sobol effect indices which quantify the variance in predictions of relative reduction in clinical incidence. These indices can be interpreted as the proportion of variation in the outcome attributed to changes in each variable. Results are shown over different parameter ranges for initial efficacy against infection (75% to 100%) and half-life duration (six to18 months), where vaccination coverage was fixed at 100%. Results are shown for a PEV co-administered with a blood stage clearance drug, for the zero- to six-, six- to 12-, 12- to 18- and 18- to 24-month periods following primary vaccination in the mass vaccination deployment schedule, for a setting with constant transmission, in settings with a 30% likelihood of accessing curative treatment within 14 days of symptom onset.

1. Vaccine and chemoprevention properties estimated using clinical trial data

**Fig M: Estimates for dose 4 and dose 5 boosting vaccine efficacy decay and SMC decay profile with 95% confidence intervals**

A) RTS,S efficacy against infection decay profile for a five-dose schedule seasonal delivery as estimated from fitting clinical trial data. The fourth dose is given 12 months following the third dose and the fifth dose is given 24 months after the primary series. B) SP+AQ efficacy decay profile estimated from fitting clinical trial data. Results are shown for best fit parameters for dose 4 and dose 5 boosting efficacy against infection from aggregated data for Burkina Faso (high prevalence and seasonal transmission) and Mali (moderate prevalence and highly seasonal transmission). The shaded regions show the 95% confidence intervals as estimated from the Bayesian optimization approach described in the methods section. RTS,S has high initial vaccine efficacy against infection of 91.1% [95% CI 74.5–99.7%] following the three-dose primary vaccination, with a biphasic immunity decay profile with a shape parameter k = 0.69, and a half-life duration (time until initial efficacy against infection decays by 50%) 7.32 months [95% CI 6–10 months]. The half-life duration is assumed to be similar for both dose 4 and dose 5.

**References**

1. Smith T, Killeen GF, Maire N, Ross A, Molineaux L, Tediosi F, et al. Mathematical modeling of the impact of malaria vaccines on the clinical epidemiology and natural history of Plasmodium falciparum malaria: Overview. Am J Trop Med Hyg. 2006;75: 1–10. doi:10.4269/ajtmh.2006.75.2_suppl.0750001

2. Smith T, Maire N, Ross A, Penny M, Chitnis N, Schapira A, et al. Towards a comprehensive simulation model of malaria epidemiology and control. Parasitology. 2008;135: 1507–1516. doi:10.1017/S0031182008000371

3. Chitnis N, Hardy D, Smith T. A Periodically-Forced Mathematical Model for the Seasonal Dynamics of Malaria in Mosquitoes. Bull Math Biol. 2012;74: 1098–1124. doi:10.1007/s11538-011-9710-0

4. Reiker T, Golumbeanu M, Shattock A, Burgert L, Smith TA, Filippi S, et al. Emulator-based Bayesian optimization for efficient multi-objective calibration of an individual-based model of malaria. Nat Commun. 2021;12: 7212. doi:10.1038/s41467-021-27486-z

5. Golumbeanu M, Yang G-J, Camponovo F, Stuckey EM, Hamon N, Mondy M, et al. Leveraging mathematical models of disease dynamics and machine learning to improve development of novel malaria interventions. Infect Dis Poverty. 2022;11: 61. doi:10.1186/s40249-022-00981-1

6. Smith T, Maire N, Dietz K, Killeen GF, Vounatsou P, Molineaux L, et al. Relationship between the entomologic inoculation rate and the force of infection for Plasmodium falciparum malaria. 2006 [cited 1 Jul 2024]. doi:10.4269/ajtmh.2006.75.2_suppl.0750011

7. Maire N, Smith T, Ross A, Owusu-Agyei S, Dietz K, Molineaux L. A model for natural immunity to asexual blood stages of Plasmodium falciparum malaria in endemic areas. Am J Trop Med Hyg. 2006;75: 19–31. doi:10.4269/ajtmh.2006.75.19

8. Collins WE, Jeffery GM. A retrospective examination of the patterns of recrudescence in patients infected with Plasmodium falciparum. Am J Trop Med Hyg. 1999;61: 44–48. doi:10.4269/tropmed.1999.61-044

9. Ross A, Killeen G, Smith T. Relationships between host infectivity to mosquitoes and asexual parasite density in Plasmodium falciparum. Am J Trop Med Hyg. 2006;75: 32–37. doi:10.4269/ajtmh.2006.75.32

10. Killeen GF, Ross A, Smith T. Infectiousness of malaria-endemic human populations to vectors. Am J Trop Med Hyg. 2006;75: 38–45. doi:10.4269/ajtmh.2006.75.2_suppl.0750038

11. Ross A, Maire N, Molineaux L, Smith T. An epidemiologic model of severe morbidity and mortality caused by Plasmodium falciparum. Am J Trop Med Hyg. 2006;75: 63–73. doi:10.4269/ajtmh.2006.75.63

12. Smith T, Ross A, Maire N, Rogier C, Trape J-F, Molineaux L. An epidemiologic model of the incidence of acute illness in Plasmodium falciparum malaria. Am J Trop Med Hyg. 2006;75: 56–62. doi:10.4269/ajtmh.2006.75.56

13. Ross A, Smith T. The effect of malaria transmission intensity on neonatal mortality in endemic areas. Am J Trop Med Hyg. 2006;75: 74–81. doi:10.4269/ajtmh.2006.75.74

14. Ekström AM, Clark J, Byass P, Lopez A, De Savigny D, Moyer CA, et al. INDEPTH Network: contributing to the data revolution. Lancet Diabetes Endocrinol. 2016;4: 97. doi:10.1016/S2213-8587(15)00495-7

15. Stuckey EM, Smith T, Chitnis N. Seasonally dependent relationships between indicators of malaria transmission and disease provided by mathematical model simulations. PLoS Comput Biol. 2014;10: e1003812. doi:10.1371/journal.pcbi.1003812

16. Tediosi F, Maire N, Smith T, Hutton G, Utzinger J, Ross A, et al. An approach to model the costs and effects of case management of Plasmodium falciparum malaria in sub-saharan Africa. Am J Trop Med Hyg. 2006;75: 90–103. doi:10.4269/ajtmh.2006.75.90

17. Gatton ML, Chitnis N, Churcher T, Donnelly MJ, Ghani AC, Godfray HCJ, et al. The importance of mosquito behavioural adaptations to malaria control in Africa. Evol Int J Org Evol. 2013;67: 1218–1230. doi:10.1111/evo.12063

18. Korenromp E, Mahiané G, Hamilton M, Pretorius C, Cibulskis R, Lauer J, et al. Malaria intervention scale-up in Africa: effectiveness predictions for health programme planning tools, based on dynamic transmission modelling. Malar J. 2016;15: 417. doi:10.1186/s12936-016-1461-9

19. Yukich JO, Chitnis N. Modelling the implications of stopping vector control for malaria control and elimination. Malar J. 2017;16: 411. doi:10.1186/s12936-017-2051-1

20. Penny MA, Verity R, Bever CA, Sauboin C, Galactionova K, Flasche S, et al. Public health impact and cost-effectiveness of the RTS,S/AS01 malaria vaccine: a systematic comparison of predictions from four mathematical models. Lancet Lond Engl. 2016;387: 367–375. doi:10.1016/S0140-6736(15)00725-4

21. Camponovo F, Ockenhouse CF, Lee C, Penny MA. Mass campaigns combining antimalarial drugs and anti-infective vaccines as seasonal interventions for malaria control, elimination and prevention of resurgence: a modelling study. BMC Infect Dis. 2019;19: 920. doi:10.1186/s12879-019-4467-4

22. Smith T, Ross A, Maire N, Chitnis N, Studer A, Hardy D, et al. Ensemble Modeling of the Likely Public Health Impact of a Pre-Erythrocytic Malaria Vaccine. PLOS Med. 2012;9: e1001157. doi:10.1371/journal.pmed.1001157

23. Chandramohan D, Zongo I, Sagara I, Cairns M, Yerbanga R-S, Diarra M, et al. Seasonal Malaria Vaccination with or without Seasonal Malaria Chemoprevention. N Engl J Med. 2021;385: 1005–1017. doi:10.1056/NEJMoa2026330

24. Cairns M, Barry A, Zongo I, Sagara I, Yerbanga SR, Diarra M, et al. The duration of protection against clinical malaria provided by the combination of seasonal RTS,S/AS01E vaccination and seasonal malaria chemoprevention versus either intervention given alone. BMC Med. 2022;20: 352. doi:10.1186/s12916-022-02536-5

25. Penny MA, Pemberton-Ross P, Smith TA. The time-course of protection of the RTS,S vaccine against malaria infections and clinical disease. Malar J. 2015;14: 437. doi:10.1186/s12936-015-0969-8

26. RTS,S Clinical Trials Partnership. Efficacy and safety of RTS,S/AS01 malaria vaccine with or without a booster dose in infants and children in Africa: final results of a phase 3, individually randomised, controlled trial. Lancet Lond Engl. 2015;386: 31–45. doi:10.1016/S0140-6736(15)60721-8

27. Thompson HA, Hogan AB, Walker PGT, White MT, Cunnington AJ, Ockenhouse CF, et al. Modelling the roles of antibody titre and avidity in protection from Plasmodium falciparum malaria infection following RTS,S/AS01 vaccination. Vaccine. 2020;38: 7498–7507. doi:10.1016/j.vaccine.2020.09.069

28. White MT, Verity R, Griffin JT, Asante KP, Owusu-Agyei S, Greenwood B, et al. Immunogenicity of the RTS,S/AS01 malaria vaccine and implications for duration of vaccine efficacy: secondary analysis of data from a phase 3 randomised controlled trial. Lancet Infect Dis. 2015;15: 1450–1458. doi:10.1016/S1473-3099(15)00239-X

29. Balandat M, Karrer B, Jiang D, Daulton S, Letham B, Wilson AG, et al. BoTorch: A Framework for Efficient Monte-Carlo Bayesian Optimization. Advances in Neural Information Processing Systems. Curran Associates, Inc.; 2020. pp. 21524–21538. Available: https://proceedings.neurips.cc/paper/2020/hash/f5b1b89d98b7286673128a5fb112cb9a-Abstract.html

30. Paszke A, Gross S, Massa F, Lerer A, Bradbury J, Chanan G, et al. PyTorch: An Imperative Style, High-Performance Deep Learning Library. ArXiv. 2019 [cited 23 Jul 2024]. Available: https://www.semanticscholar.org/paper/PyTorch%3A-An-Imperative-Style%2C-High-Performance-Deep-Paszke-Gross/3c8a456509e6c0805354bd40a35e3f2dbf8069b1

31. Wilson JT, Moriconi R, Hutter F, Deisenroth MP. The reparameterization trick for acquisition functions. arXiv; 2017. doi:10.48550/arXiv.1712.00424
